# Hair Shaft Examination: A Practical Tool to Diagnose Griscelli Syndrome

**DOI:** 10.3390/dermatopathology8010010

**Published:** 2021-03-09

**Authors:** Trinidad Montero-Vilchez, Alexandra Remon-Love, Jesús Tercedor-Sánchez, Salvador Arias-Santiago

**Affiliations:** 1 Department of Dermatology, Hospital Universitario Virgen de las Nieves, 18012 Granada, Spain; tmonterov@correo.ugr.es (T.M.-V.); salvadorarias@ugr.es (S.A.-S.); 2 Department of Pathology, Hospital Universitario Virgen de las Nieves, 18012 Granada, Spain; a-remon@hotmail.com

**Keywords:** Griscelli syndrome, hair shaft, silvery

## Abstract

Griscelli syndrome (GS) is a rare disease that is characterized by silvery hair and fair skin. It is included in congenital grey hair syndromes, a rare group of autosomal recessive disorders characterized by silvery grey hair and severe multisystem disorders, such as immune system impairment, defects in immunological function, ocular and skeletal alterations, and nervous system defects. Herein, we report a rare case of GS type 1 and highlight the importance of a dermatological and hair examination to make an early diagnosis of these life-threatening diseases.

## 1. Introduction

Griscelli syndrome (GS) is a rare skin disease characterized by a silvery hair and cutaneous hypopigmentation [1]. Three types of GS have been described. GS type 1 is characterized by hypomelanosis and primary neurological deficit [2]. GS type 2 manifests as hypomelanosis and immune deterioration [3]. GS type 3 only shows skin manifestations [4]. This syndrome starts between infancy and childhood. Besides shiny hair and fair skin, patients with GS type 1 have delayed motor development, intellectual disability, and hypotonia [2]. Patients with GS type 2 tend to have an associated cytotoxic lymphocyte defect, resulting in a hemophagocytic syndrome of uncontrolled macrophage and T-lymphocyte activation. Activated lymphocytic T cells and macrophages infiltrate lymph nodes and other organs (including the brain), leading to a hemophagocytosis phenomenon [3].

GS type 1 is caused by mutations in the MYO5A gene located on chromosome 15q21, GS type 2 is caused by mutations in the RAB27A (15q21.3) gene, and GS type 3 is caused by mutations in the MLPH (2q37.3) gene [1,5,6]. GS type 1 inheritance is autosomal recessive. This means that if both parents were heterozygous carriers for the pathogenic mutation, the theoretical risk for the offspring would be 25% homozygous affected, 50% asymptomatic heterozygous carrier, and 25% non-affected non-carrier [2].

## 2. Case Report

A 10-year-old Spanish girl presented to our dermatologic clinic with discoloration of the scalp and eyebrow since birth. She was born out of consanguineous marriage and had a twin sister with similar phenotype. A 13-year-old brother was healthy. The patient’s clinical history revealed that she also suffered from developmental delay and epilepsy with tonic-clonic seizures. Moreover, she had severe neuromuscular scoliosis, with perpendicular obliquity of sapropelic origin. She also suffered from gastro-oesophageal reflux and was being studied by an endocrinologist for early puberty.

**Figure 1:**
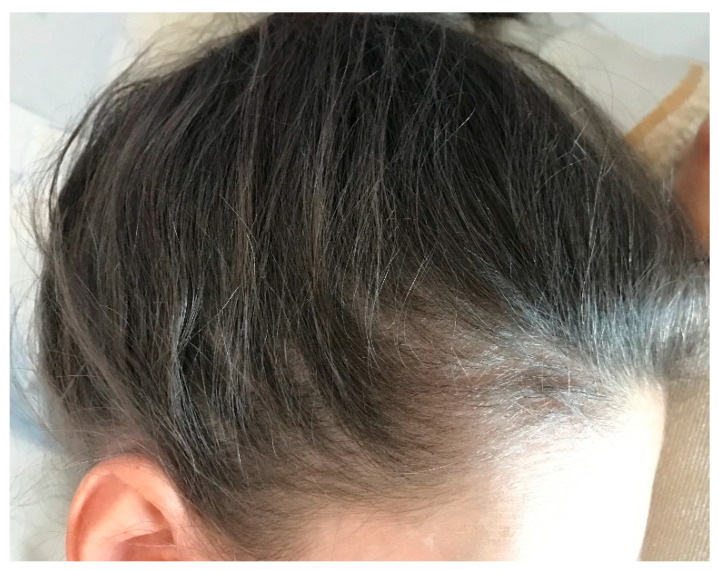
Silvery grey scalp hair.

**Figure 2:**
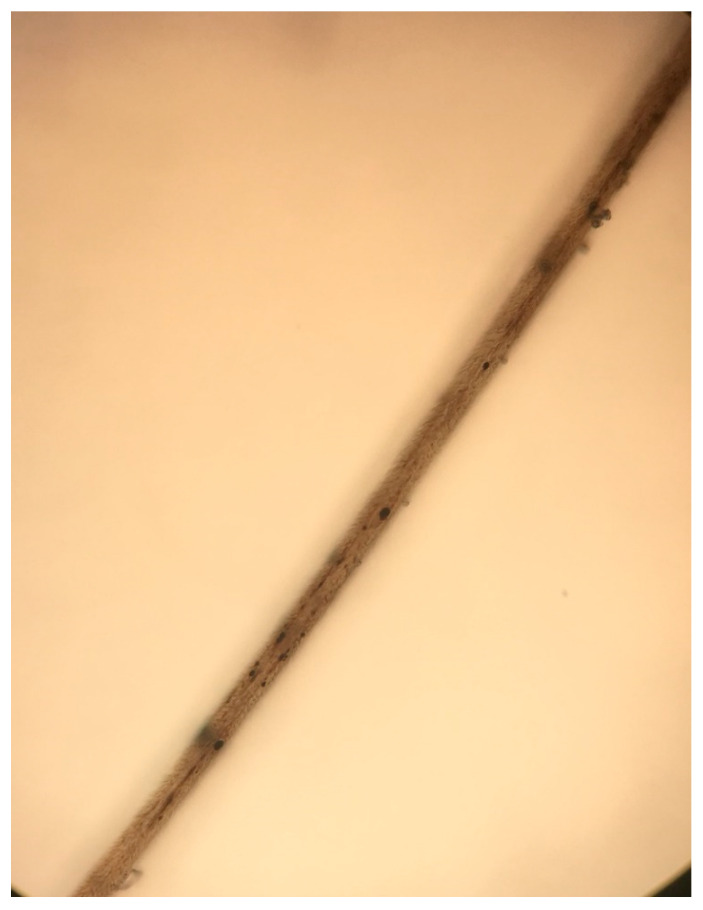
Microscopic examination of a hair shaft.

**Figure 3:**
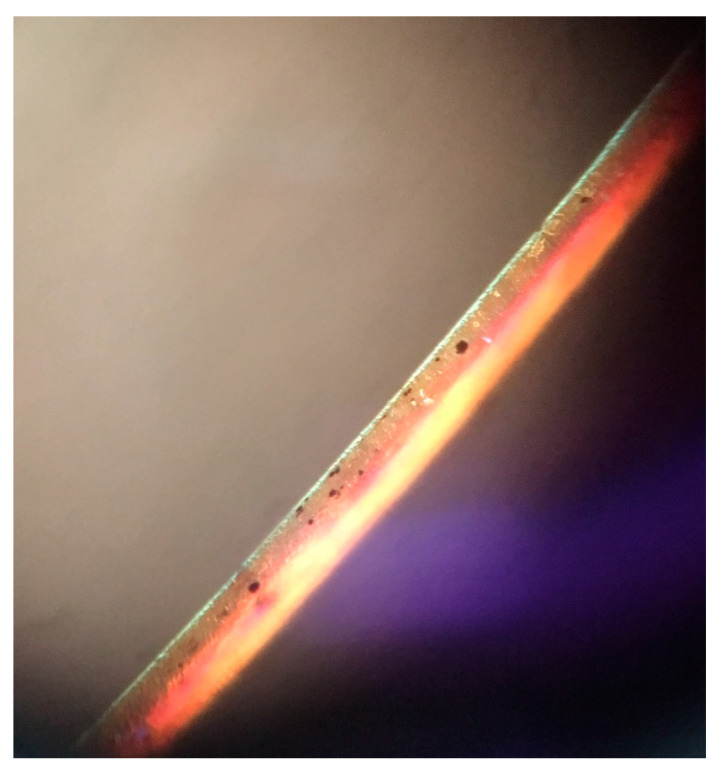
Hair shaft showing large irregular melanin granules.

The physical examination showed facial hypopigmentation, greyish eyebrows, and grey hair tufts (Figure 1). Laboratory studies showed normal results. Hair shaft examination under light microscopy showed giant uneven melanin granules in the medullar zone (Figure 2 and Figure 3), a typical characteristic observed in Griscelli syndrome (GS). So, regarding clinical manifestation and microscopy features, the patient was diagnosed with GS type 1. Following ethics approval and informant consent, DNA was extracted from peripheral blood samples. The diagnosed of GS type 1 was then confirmed, as the patient was a homozygous carrier of the pathogenic variant c.5152C>T (p.Gln1718*) in the MYO5A gene NM_000259.3.

## 3. Discussion

GS is included in congenital grey hair syndromes, a rare group of autosomal recessive disorders characterized by silvery grey hair, defects in immunological function, and nervous system defects. Besides GS, Chediak–Higashi syndrome (CHS), Elejalde syndrome (ES), and oculocerebral hypopigmentation syndrome Cross type (OHS) are also included [6]. It was previously mentioned that GS type 1 is characterized by hypomelanosis and primary neurological deficit [2]. GS type 2 manifests as hypomelanosis and immune deterioration [3]. GS type 3 only shows skin manifestations [4]. CHS is characterized by severe cell immunodeficiency and oculocutaneous albinism with silver hair [7]. ES manifests with neurologic defects and ocular signs such as nystagmus, diplopia, or congenital amaurosis [8]. OHS is characterized by neurological signs, growth deficiency, intellectual disability, and ocular signs such as microphthalmia or ectropium [9].

Clinically, it is difficult to distinguish these disorders, as their clinical features may overlap. Nevertheless, the clinical history, physical examination, and hair shaft examination could provide a rapid clinical suspicion of this severe disease. Moreover, GS and CHS can be differentiated by hair microscopy [10]. Large irregular melanin granules are observed in all types of GS, while small melanin granules homogeneously distributed are shown in CHS [11].

Dermatopathology in GS is characterized by enlarged hyperpigmented basal melanocytes with sparsely pigmented adjacent keratinocytes. Large pigment clumps in the medullary area of hair shafts with an intermittent distribution, similar to a road-dividing line, are observed in hair shaft examination [12]. Perinuclear aggregation of melanosomes within melanocytes can be found using confocal microscopy in patients with GS type 3 [13]. Electron microscopy shows uneven clusters of aggregated melanin pigment in hair shaft and type IV melanosomes and shortened dendritic processes among basilar melanocytes [14]. Histology in CHS is characterized by a reduction or even absence of melanin pigment in hair follicles and the basal layer, and the presence of few large pigment granules, corresponding to giant melanosomes [15]. Light microscopic shows evenly distributed melanin granules of regular diameter, larger than those of normal hair [16]. Large cytoplasmic inclusions in cutaneous mast cells are observed with toluidine blue staining [17], and giant melanosomes and degenerating cytoplasmic residues in melanocytes are found with electron microscopy [18]. Histopathology in ES is characterized by melanin granules in the basal layer with irregular size and distribution and overall reduced pigmentation. Hair shafts are like those seen in GS. ES also shows abnormal inclusion bodies in fibroblasts, dermal collagenization, and subcutaneous edema [8,19].

Herein, this report highlights hair shaft examination under light microscopy as a cheap, easy, non-invasive, time-saving diagnostic tool that may help clinicians to promptly identify these life-threatening congenital diseases.
